# COVID-19 variant radiological findings with high lightening other coronavirus family (SARS and MERS) findings: radiological impact and findings spectrum of corona virus (COVID-19) with comparison to SARS and MERS

**DOI:** 10.1186/s43055-020-00262-7

**Published:** 2020-08-28

**Authors:** Marian Fayek Kolta, Mai Bahgat Ibrahim Ghonimy

**Affiliations:** Radiology Department, Thoracic Imaging Unit, Faculty of medicine, Kasr Al-Aini, Cairo University, Cairo, Egypt

## Abstract

**Background:**

Chest CT is remarkably considered as an imminent diagnostic tool and follow-up study in pulmonary changes in COVID-19 patients; being familiar to other coronavirus family CT findings, this improve our diagnostic experience and hence enhance our ability to early diagnose and combat the outbreak of COVID-19. The purpose is to investigate the wide spectrum of radiological pulmonary changes in COVID-19 patients and compare them to the variable CT findings reported in MERS and SARS.

**Results:**

From March 15 to May 12, 2020, 50 patients in Cairo, Egypt, who have positive RT-PCR tests, were included in our study. MSCT of the chest was performed to all patients and processed in a separate work station. Two experienced radiologists assessed each study for the type and location of different pulmonary affection.

The most imminent radiological finding was patchy peripheral subpleural ground glass opacity found in 42 patients (84% of cases), followed by consolidation found in 30 patients (60% of cases) and ground glass and consolidation together found in 22 patients (44% of cases).

Unlike SARS, where initial chest imaging abnormalities are more frequently unilateral, COVID-19 is more likely to involve both lungs on initial imaging presented as bilateral peripheral subpleural scattered ground-glass opacities.

Pleural effusion is absent in COVID-19 patients while it is not rare in MERS and might be observed in 20–33% of affected individuals.

**Conclusion:**

The imaging features of COVID-19 pneumonia are highly sensitive mainly in the outbreak pandemic.

The imaging features of SARS, MERS, and COVID-19 overlap, but differences still exist especially early in disease course.

## Background

The 2019 novel coronavirus (2019-nCoV) is a new pandemic disease diagnosed at the late 2019 in China, Wuhan. Chest CT is a key component of the diagnostic work-up for patients with suspected infection [1].

Radiological examinations are vital in early diagnosis and assessment of disease course, as most COVID-19 infected patients were diagnosed with pneumonia and characteristic CT imaging patterns [1].

In absence of specific therapeutic drugs or vaccines for 2019 novel coronavirus disease (COVID-19), it is essential to detect the diseases at an early stage and immediately isolate the infected person from the healthy population [2].

The low sensitivity of RT-PCR implies that many COVID-19 patients may not be identified and may not receive appropriate treatment in time; such patients constitute a risk for infecting a larger population given the highly contagious nature of the virus [3].

Chest CT, as a routine imaging tool for pneumonia diagnosis, is relatively easy to perform and can produce fast diagnosis [4].

Investigators are making every effort to further characterize the imaging features of this novel coronavirus syndrome, but information is still limited [1].

Chest CT is a conventional, non-invasive imaging modality with high accuracy and speed. Based on available data published in recent literature, almost all patients with COVID-19 had characteristic CT features in the disease process [2].

The chest CT scans showed a higher sensitivity for the diagnosis of COVID-19 infection than initial RT-PCR results [2].

Similar pulmonary syndromes have been recognized as being caused by other strains of the coronavirus family. The most striking examples are the severe acute respiratory syndrome (SARS) and the Middle East respiratory syndrome (MERS) [5].

Imaging is a critical component of the diagnostic workup, monitoring of disease progression, and follow-up in coronavirus-related pulmonary affection [6].

Since the etiologic and clinical features of the syndrome are similar to those of SARS and MERS, the experience from those pulmonary syndromes can be helpful for managing the emerging COVID-19 outbreak [5].

The aim of this study is to familiarize radiologists with the imaging spectrum of coronavirus syndromes and to discuss the reported imaging features of COVID-19 comparing them to SARS and MERS findings.

## Methods

This cross section study included 50 patients (43 males, 7 females) with age range from 32 to 75 years (mean age of 47.2 years) confirmed to be infected with SARS-CoV-2, referred for multislice CT (MSCT) assessment of the chest (Table 1). MSCT of the chest was done to all patients as requested. The study was conducted between March 15 and May 12, 2020, in Cairo, Egypt.

**Table 1:** Parameters of MSCT of the chest

Tube voltage	120 kVp
Tube current	60–120 mAp
Slice thickness	1 mm
Reconstruction interval	1 mm
Patient position	Supine
Respiration	Breath hold full inspiration
Matrix size	512 × 512

### Inclusion criteria

The inclusion criterion is laboratory proven PCR positive COVID-19 tests.

### Exclusion criteria

The exclusion criteria are as follows:

Pregnant females

Patients presenting with acute heart failure

Patients who recently experienced clinically defined pulmonary infection attributable to other pathogens

Patients with severe artifacts on CT images

### Methods

All enrolled patients were subjected to as follows:

- ❖ Through history taking.
- ❖ Laboratory assessment (CBC, ESR, and PCR).

MSCT of the chest was done to all patients using a multi-detector CT scanner with 64 or more channels. The detailed parameters for CT acquisition were as follows: tube voltage, 120 kVp; tube current, standard (reference mAs, 60–120) to low dose (reference mAs, 30) with automatic exposure control; slice thickness, 1.0 mm; reconstruction interval, 1.0–3.0 mm; and a sharp reconstruction kernel. CT images were obtained with the patient in the supine position at full inspiration, head first and without contrast medium (Table 2).

- ❖ Then, the images acquired sent to a separate workstation to be processed, manipulated, and reconstructed.
- ❖ Images are reconstructed in axial, coronal, and sagittal planes to detect the distribution of parenchymal affection (2D multiplanar images reconstruction, MPR).
- ❖ All images were viewed on both lungs (width, 1500 HU; level, − 700 HU) and mediastinal (width, 350 HU; level, 40 HU) settings.
- ❖ For each patient, the chest CT scan was evaluated by two radiologists separately searching for the following characteristics: (1) presence of ground-glass opacities, (2) presence of consolidation, (3) laterality of ground-glass opacities and consolidation, (4) presence of nodules, (5) presence of a pleural effusion, (6) presence of thoracic lymphadenopathy (defined as lymph node size of ≥ 10 mm in short-axis dimension), (7) airways abnormalities (including airway wall thickening, bronchiectasis, and endoluminal secretions), (8) axial distribution of disease (categorized as no axial distribution of disease, central “peribronchovascular” predominant disease, or peripheral predominant disease), and (9) other abnormalities, including linear opacities, opacities with a rounded morphology, opacities with a “reverse halo” sign, and opacities with a “crazy-paving” pattern.

**Table 2:** Different spectrum of radiological features

Radiological feature	Number of patients	Percent of patients (%)
Ground glass	42	84
Consolidation	30	60
Ground glass and consolidation	22	44
Bilateral affection	38	76
Peripheral affection	42	84
Coarse basal pulmonary interstitium	8	16
Basal curvilinear atelectasis	7	14
Reversed halo sign	3	6
Crazy paving pattern	3	6
Mild bronchiectatic	2	4

### Statistical analysis

Findings are presented as medians, and interquartile ranges due to small sample size categorical variables are described as whole numbers, with percentages in brackets.

## Results

This cross section study included 50 patients (43 males, 7 females) with age ranging from 32 to 75 years (mean age of 47.2 years), with PCR positive COVID results. They were referred to perform MSCT of the chest.

Most patients presented with dyspnea, which was seen in 43 patients (86%), 40 patients suffered from fever (80%), and 25 patients presented with dry cough (50%).

The most imminent radiological finding was ground-glass opacity found in 42 patients (84% of cases), followed by consolidation found in 30 patients (60% of cases) and ground glass and consolidation together found in 22 patients (44% of cases) as shown in Table 2.

Twenty-two patients showed lower zone predominance (44%), 18 patients showed equal distribution between the upper and lower zones (36%), and ten patients showed upper zone predominant changes (20%) (Table 3).

**Table 3:** Predominant distribution

Predominant distribution	Number of cases	Percent (%)
Peripheral	42	84
Peri-hilar	2	4
Diffuse	6	12
Upper lobar	18	44
Lower lobar	22	36
Upper and lower lobar	10	20

The ground-glass and consolidative opacities were peripheral in most patients with lung findings (*n* = 42), while 6 patients who had diffuse ground-glass changes and 2 patients showed peri-hilar distribution (Table 3).

Other less common findings like coarse pulmonary interstitium were in 8 patients (16%); basal curvilinear atelectasis was seen in 7 patients (14%); reversed halo sign was seen in three patients (6% of cases); crazy paving pattern (6%) and mild bronchiectatic changes were noticed in 2 patients (4% of cases).

Pleural effusions, pericardial effusion, cavitation, mediastinal, and hilar lymph node enlargement were not seen in any of the patients.

## Discussion

Lower respiratory tract infections are the most lethal transmissible diseases worldwide, causing around 3 million deaths per year [7].

Previously, six types of coronavirus had been identified that cause human disease: four cause mild respiratory symptoms, whereas the other two, Middle East respiratory syndrome (MERS) coronavirus and severe acute respiratory syndrome (SARS) coronavirus, have caused epidemics with high mortality rates [7].

In 2019, a new strain, called SARS-CoV-2, started circulating all over the word as a pandemic, causing the disease COVID-19 [6].

Imaging is confirmed to be critical in assessing severity and disease progression in COVID-19 infection [4].

Variety of imaging features seen while studying MSCT of chest of COVID-19 patients shows great similarity to that described in other coronavirus-associated syndromes.

Sensitivity and specificity of chest CT for COVID-19 are reported to range from 80 to 90% and 60 to 70%, respectively [8, 9].

In our study, we noted dyspnea was the commonest clinical symptoms which was in disagreement with the study done by Andrea et al. [10] who noted that fever was the most common symptoms in COVID positive patients, followed by cough and fatigue. Fever is 85.6%, cough is 68.7%, and fatigue is 39.4%.

**Fig. 1:**
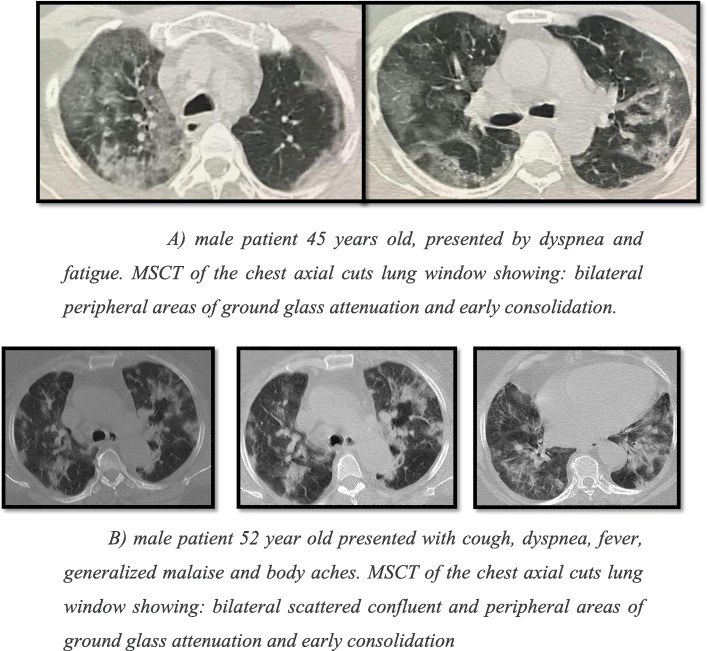
**a** Male patient, 45 years old, presented by dyspnea and fatigue. MSCT of the chest axial cuts the lung window showing bilateral peripheral areas of ground-glass attenuation and early consolidation. **b** Male patient, 52 years old, presented with cough, dyspnea, fever, generalized malaise, and body aches. MSCT of the chest axial cuts the lung window showing bilateral scattered confluent and peripheral areas of ground-glass attenuation and early consolidation

According to the different radiological findings in the study population, it was noted that ground-glass opacification was the most common radiological finding 84%, followed by consolidation 60% which agrees with Ming et al. [11] who studied the imaging profile of the COVID-19 infection and agrees with Melina et al. [5] which showed that multifocal ground-glass opacities and consolidation were reported as main radiological features (Fig. 1).

**Fig. 2:**
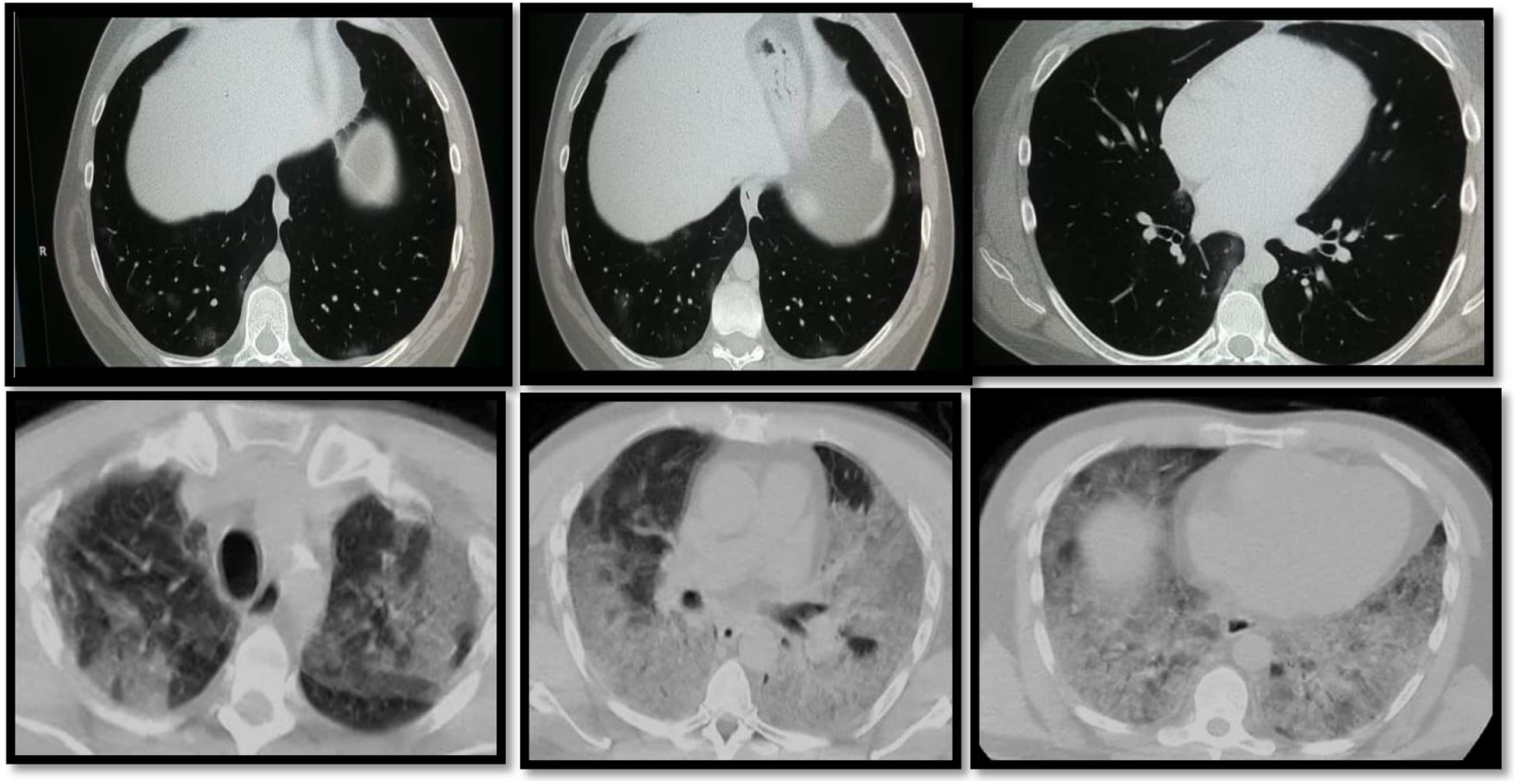
A male patient, 53 years old, presented with dyspnea. MSCT chest lung window was done showing bilateral few faint mainly basal ground-glass nodular peripheral shadows. Two days later, the patient deteriorated clinically suffering severe dyspnea and fever. A follow-up MSCT chest was done showing bilateral diffuse ground glass and consolidative patches in both lung fields

**Fig. 3:**
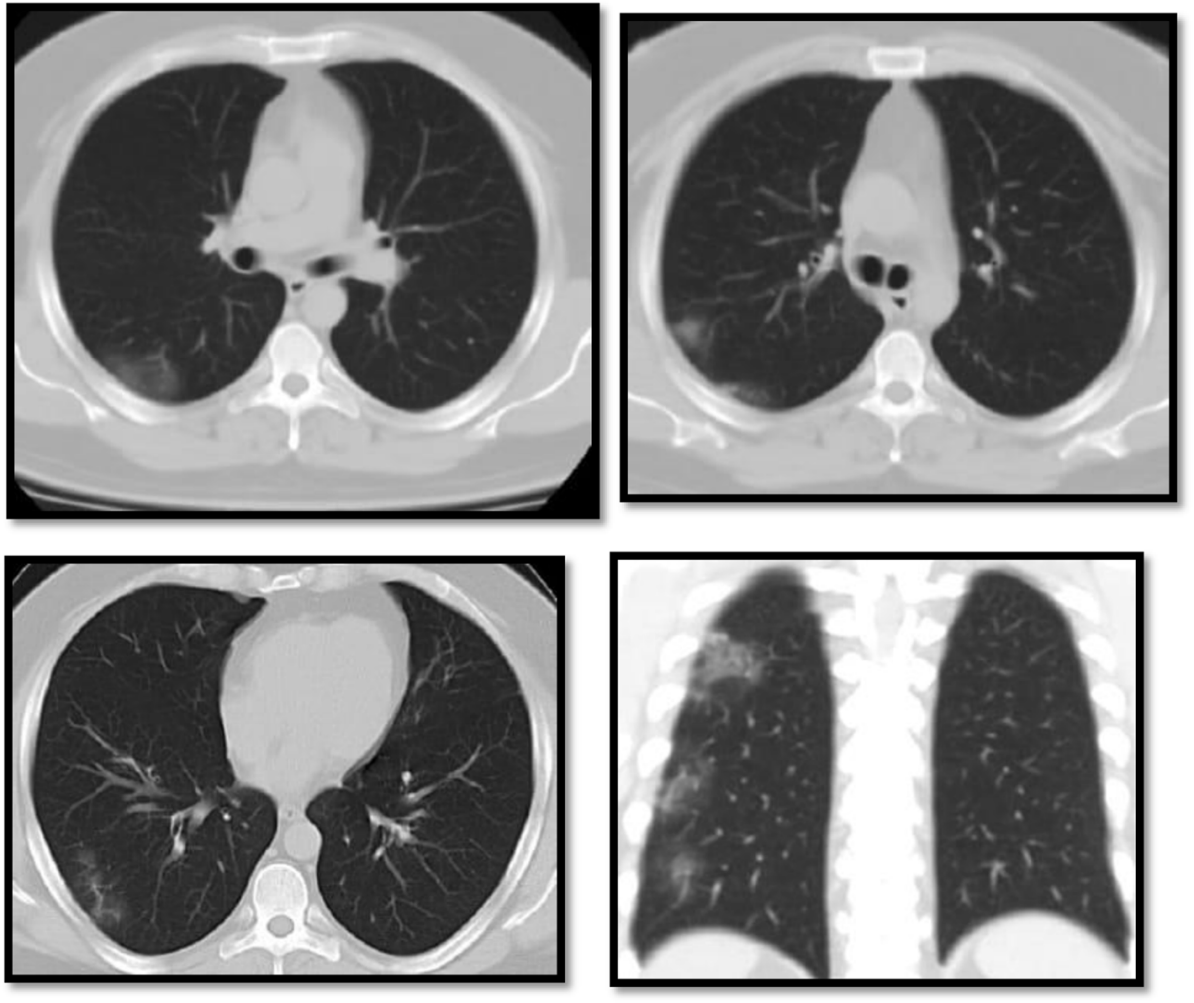
A female patient, 37 years old, resented with fever and cough. MSCT of the chest was done showing typical peripheral distribution of the ground-glass opacities seen in COVID positive patients

The ground-glass and consolidative opacities were peripheral in most patients with lung findings (*n* = 42, 84%), while 6 patients had diffuse ground-glass changes and 2 patients showed peri-hilar distribution, which is matching with a study done by Ming et al. [11] who found that lung affection was peripheral in all patients with lung findings (100%), apart from one patient who had peri-hilar ground-glass changes in addition (Figs. 2 and 3).

Twenty-two patients showed lower zone predominance (44%), 18 patients showed equal distribution between the upper and lower zones (36%), and ten patients showed upper zone predominant changes (20%), which is partially agreeing with Ming’s study [11] who found that 44% of patients showed lower zone predominance, while 44% of patients showed equal distribution between the upper and lower zones and 16% of patients showed upper zone predominant changes and agrees also to the study done by Ho et al. [12], who demonstrated that the common CT findings of bilateral involvement, peripheral distribution, and lower zone dominance.

**Fig. 4:**
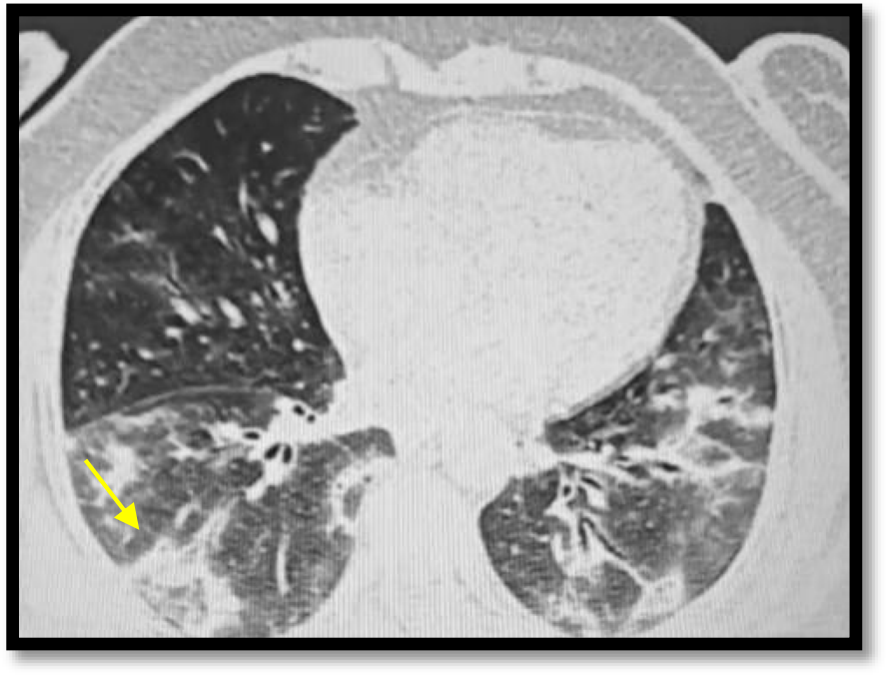
A male patient, 48 years old, presented with dyspnea and fatigue. MSCT of the chest was done showing bilateral peripheral patchy areas of ground-glass attenuation, with one of them showings characteristic reversed halo sign (arrowed)

Reversed halo sign was seen in 3 cases (6%) which is agreeing with Ming et al. who stated that reversed halo sign was seen infrequently (Fig. 4).

**Fig. 5:**
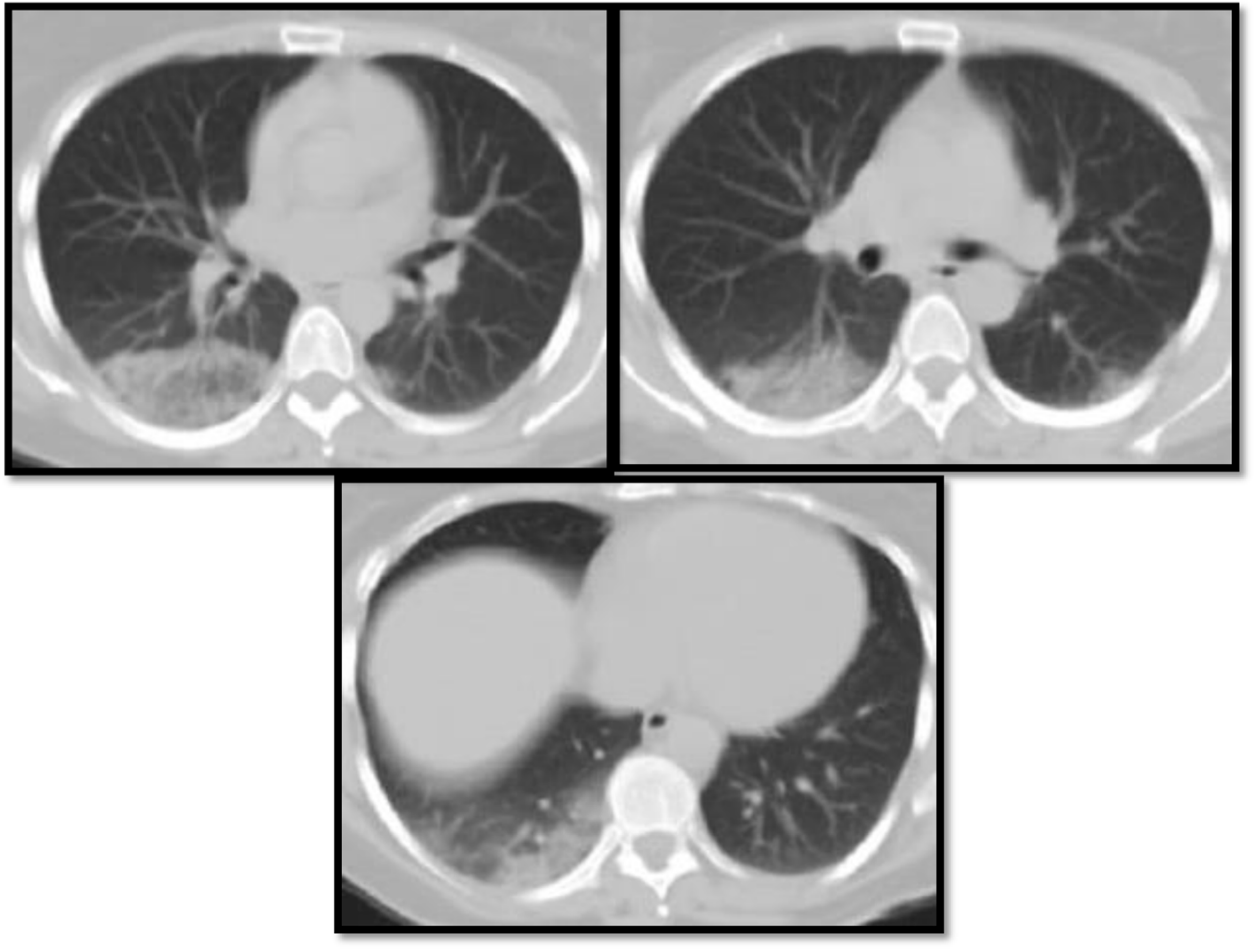
A male patient, 55 years old, presented with fever and extreme fatigue. MSCT of the chest was done showing right basal coarsening of the interstitium within the ground-glass patches, which is a typical finding detected in COVID-19 viral infection

We found coarsening of basal pulmonary interstitium in 8 cases (16%) which does not match with the study done by Shuchang et al. [13] who found GGO plus a reticular pattern in 62.9% of his patients (Fig. 5).

**Fig. 6:**
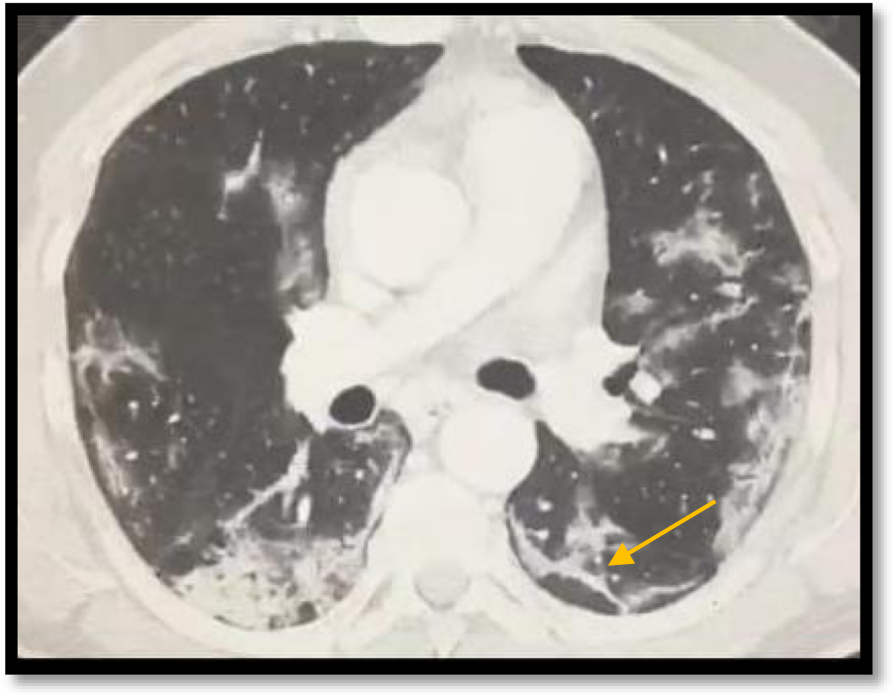
Male patient, 35 years old, presented positive for COVID infection showing bilateral middle lung zones curvilinear subpleural atelectasis with peripheral consolidative patches

Curvilinear subpleural opacity was seen in 7 patients (14%) which is matching with studies by Wu et al. [14] and Li et al. [15] both reported around 20% of patients with COVID-19 demonstrated this sign, which might relate to pulmonary edema or fibrosis of COVID-19 (Fig. 6).

**Fig. 7:**
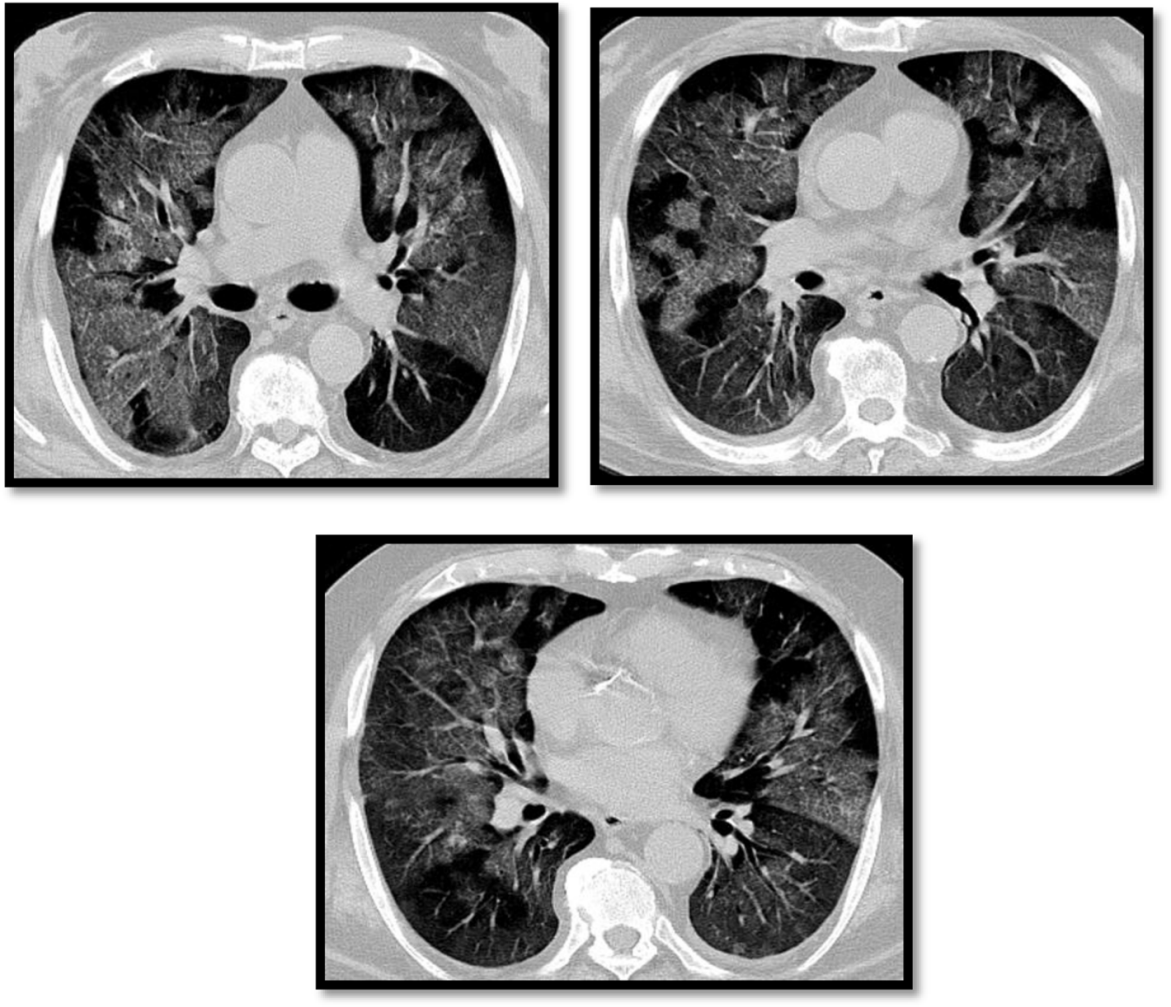
A male patient, 48 years old, presented by severe respiratory distress. MSCT was done showing bilateral diffuse patchy ground-glass opacification with superimposed prominent intralobular septae giving crazy paving pattern

Crazy paving pattern was seen in 3 patients (6%) which is matching with many recent investigations reported 5~36% COVID-19 patients with crazy paving pattern in their studies [15, 16]. Furthermore, the presence of diffuse GGO, consolidation, and crazy paving pattern can be the signal of COVID-19 entering progressive or peak stage [17] (Fig. 7).

Pleural effusions, pericardial effusion, cavitation, mediastinal, and hilar lymph node enlargement were not seen in any of our patients which agree with Ming’s study [11] which declared that lung cavitation, discrete pulmonary nodules, pleural effusions, and enlarged lymph nodes were absent.

Since COVID/SARS and MERS are considered from the same viral family (coronavirus), imaging features of COVID-19, SARS, and MERS overlap, but still differences exist as well.

Unlike SARS, where initial chest imaging abnormalities are more frequently unilateral, COVID-19 is more likely to involve both lungs on initial imaging presented as bilateral peripheral subpleural scattered ground-glass opacities [5]. The majority of SARS positive patients show progressive multifocal distribution, in the follow-up imaging, of which 75% of patients show bilateral distribution [18], while MERS initial imaging tends to show multifocal airspace opacities in the lower lung zones which then progress to extend peri-hilar and upper lobar [19].

Pleural effusion is absent in COVID-19 patients while it is not rare in MERS and might be observed in 20–33% of affected individuals [5].

Centrilobular nodules and tree-in-bud are not characteristics of SARS or MERS [20], which is the same in COVID-19 according to our study.

Overall, the imaging findings are highly sensitive yet highly nonspecific and might overlap with the symptoms of H1N1 influenza, cytomegalovirus pneumonia, or atypical pneumonia. The acute clinical presentation and history of contact with a COVID-19-infected patient or history of recent travel should raise clinical suspicion for the diagnosis of COVID-19 [21].

## Conclusion

The imaging features of COVID-19 pneumonia are highly sensitive yet nonspecific and are more often bilateral with subpleural and peripheral distribution and range from ground-glass opacities in milder forms to consolidations in more severe forms.

The imaging features of SARS, MERS, and COVID-19 overlap, but differences exist especially early in disease course.

## Data Availability

Data available within the article or its supplementary materials.

## Abbreviations

MSCT: Multislice CT
COVID-19: Coronavirus disease 2019
RT-PCR: Real time polymerase chain reaction
SARS: Severe acute respiratory syndrome
MERS: Middle East respiratory syndrome
